# S1 Subunit of Spike Protein from a Current Highly Virulent Porcine Epidemic Diarrhea Virus Is an Important Determinant of Virulence in Piglets

**DOI:** 10.3390/v10090467

**Published:** 2018-08-30

**Authors:** Tohru Suzuki, Yutaka Terada, Luis Enjuanes, Seiichi Ohashi, Wataru Kamitani

**Affiliations:** 1 Division of Viral Disease and Epidemiology, National Institute of Animal Health, National Agricultural Research Organization, Tsukuba, Ibaraki 3050856, Japan; ohashis@affrc.go.jp; 2 Research Institute for Microbial Diseases, Osaka University, Suita, Osaka 5650871, Japan; yterada@biken.osaka-u.ac.jp (Y.T.); wakamita@biken.osaka-u.ac.jp (W.K.); 3 Department of Molecular and Cell Biology, Centro Nacional de Biotecnologia, Campus Universidad Autónoma de Madrid, 28049 Madrid, Spain; l.enjuanes@cnb.csic.es

**Keywords:** porcine epidemic diarrhea virus, coronavirus, spike protein, virulence, gnotobiotic piglets, reverse genetics

## Abstract

Base on the sequence of *S* genes, which encode spike proteins, we previously identified three different types (North American, S INDEL, and S large-DEL types) of porcine epidemic diarrhea virus (PEDV) that have re-emerged in Japan since 2013. Based on experimental infections with the North American and S large-DEL types, we also hypothesized that PEDV virulence may be linked to the S1 subunit of the S protein. To test this hypothesis, we have now assayed in gnotobiotic piglets various recombinant PEDVs generated by reverse genetics. Piglets inoculated with CV777 maintained in National Institute of Animal Health, along with piglets infected with a recombinant form of the same virus, developed subclinical to mild diarrhea. In contrast, severe watery diarrhea, dehydration, weight loss, astasia, and high mortality were observed in piglets inoculated with recombinant strains in which the *S* gene was partially or fully replaced with corresponding sequences from the highly virulent Japanese PEDV isolate OKN-1/JPN/2013. Indeed, symptoms resembled those in piglets inoculated with the OKN-1/JPN/2013, and were especially pronounced in younger piglets. Collectively, the data demonstrate that the S1 subunit of the S protein is an important determinant of PEDV virulence, and advance development of new vaccine candidate.

## 1. Introduction

Porcine epidemic diarrhea is characterized by severe watery diarrhea which causes dehydration and high mortality among piglets. The disease is due to porcine epidemic diarrhea virus (PEDV), which belongs to the family *Coronaviridae* and genus *Alphacoronavirus*, and has an envelope surrounding a single-stranded positive-sense RNA genome [1]. The disease was first described in the United Kingdoms in 1971 [2], and has since widely spread not only to other European countries, but also to Asian countries including China, Korea, and Japan [3,4,5,6,7,8]. Notably, severe outbreaks caused by a strain genetically distinct from the prototype strain CV777 based on analysis of *S* genes, which encode spike proteins, have been reported in China since late 2010, with considerable morbidity and mortality among suckling piglets [9,10,11,12]. In April 2013, the disease was detected for the first time in the US, and was rapidly dispersed over 30 states in just one year [13,14]. There were mainly two different types of PEDV strains in the US based on analysis of the *S* genes: the original highly virulent strain (North American type), which is genetically closely related to strains that emerged in China since 2010, and a mildly virulent strain (S INDEL type) with insertions and deletions at the 5’ end of the S gene [15,16]. US-like epidemics have also occurred in Canada and Mexico in North America, Korea and Taiwan in Asia, and Germany and Belgium in Europe [16,17,18,19,20,21,22].

In Japan, porcine epidemic diarrhea was first reported in 1982 [4]. In 1996, outbreaks occurred in 80,000 pigs at over 100 farms in nine prefectures, of which approximately half died. Although no cases were reported beginning in 2006, the disease re-emerged in October 2013. Thereafter, Ministry of Agriculture, Forestry and Fisheries (http://www.maff.go.jp) has reported over 1000 outbreaks across almost all (39/47) prefectures, killing approximately 500,000 pigs until July 2018. To investigate the cause of re-emergence, we analyzed the *S* genes in 38 PEDV strains collected from 18 prefectures between 2013 and 2014, and detected the North American type (*n* = 33), the S INDEL type (*n* = 4), and the S large-DEL type (*n* = 1), a novel variant with a large deletion of 582 nucleotides (194 amino acids) at the 5’ end [23,24]. In addition, this analysis revealed that the strains are more similar to global PEDV strains detected in recent years than to classical strains detected in Japan decades ago. Moreover, a phylogenetic dendrogram constructed from complete genomes indicated that the strains are genetically closely related to strains widespread in the US and Korea in 2013–2014, and that the S large-DEL variant may have spontaneously arisen from strains already circulating in domestic pigs [24,25]. On experimental infections with the North American or the S large-DEL type, colostrum-deprived piglets, which are highly susceptible to pathogens, developed acute severe or moderate watery diarrhea, although the former was lethal while the other was not [26]. Moreover, the strains propagated in varying fashion to different tissues and formed different intestinal lesions.

The coronavirus spike protein consists of S1 and S2 subunits [27], of which the former binds putative cellular receptors such as aminopeptidase N and sialic acid, whereas the latter mediates virus-cell membrane fusion during entry [28]. Strikingly, porcine respiratory coronavirus has a large deletion of 200–230 amino acids in the S1 subunit, and is believed to be a naturally occurring mutant of, but with different tropism and pathogenicity as transmissible gastroenteritis virus, also of the genus *Alphacoronavirus* [29,30,31]. Therefore, these observations imply that the S protein, especially the S1 subunit, may play an important role in virulence and tissue tropism.

Reverse genetics, a powerful approach to analyze the function and role of a single gene, has been also used recently in coronaviruses which have large genomes of approximately 30 Kb, including for PEDV, severe acute respiratory syndrome and Middle East respiratory syndrome coronaviruses [32,33,34,35]. To evaluate whether the *S* gene, especially the S1 subunit is an important determinant of PEDV virulence, we have now infected gnotobiotic piglets with several recombinant PEDVs that were generated by reverse genetics to fully or partially replace S genes, but otherwise retain common genetic backbones.

## 2. Material and Methods

### 2.1. Cells and Viruses

Huh7 (human liver carcinoma) and Vero (African green monkey kidney cells) cells were maintained in Dulbecco’s modified Eagle’s medium (DMEM) (Nacalai Tesque, Kyoto, Japan) supplemented with 10% fetal bovine serum (FBS), 100 U/mL penicillin, and 100 µg/mL streptomycin (Nacalai Tesque, Kyoto, Japan). All cells were cultured at 37 °C in a humidified atmosphere with 5% CO_2_. The prototype PEDV strain, CV777 was kindly provided by Dr. M.B. Pensaert and, thereafter, passaged and maintained at several times in our institute (The cells maintained in our institute were designated as the CV777-niah strain). The complete genome of the CV777-niah strain had eleven nucleotide changes including eight non-synonymous substitutions, and three deletion (1–52 nucleotide in length) as compared with that of the reference CV777 strain (GenBank accession no. AF353511) (Table 1). The highly virulent PEDV strain, OKN-1/JPN/2013 was isolated and maintained in Vero cells as described in our previous study [24].

### 2.2. Construction of Bacterial Artificial Chromosomes (BAC)

A BAC clone carrying full-length genome of the CV777-niah strain, pBAC-PEDV-CV777-FL, was generated on the backbone of SARS-CoV-Rep [36] using a Red/ET Recombination System Counter-Selection BAC Modification Kit (Gene Bridges, Heidelberg, Germany). However, the *S* gene of pBAC-PEDV-CV777-FL was modified to encode the entire spike protein based on CV777 reference sequence (GenBank accession no. AF353511). Using the same kit, *ORF3* gene was replaced with green fluorescent protein (*GFP*) gene driven by the ORF3 transcription regulatory sequence, yielding pBAC-PEDVGFP-CV777, an infectious clone expressing GFP from subgenomic RNA. Finally, this clone was modified using the same kit to generate chimeric clones in which the *S* gene is fully or partially (S1 subunit) replaced with that of a high virulent Japanese PEDV strain, OKN-1/JPN/2013 (GenBank accession no. LC063836), yielding pBAC-PEDVGFP-S-OKN1 and pBAC-PEDVGFP-S1-OKN1, respectively.

### 2.3. Recovery of Recombinant PEDVs

Huh7 cells were grown to approximately 80% confluence in a six-well plate (VIOLAMO, Osaka, Japan), and transfected with 8 μg BAC DNA using X-tremeGENE 9 DNA Transfection Reagent (Sigma-Aldrich, St. Louis, MO, USA). After 6 h, media were replaced with fresh DMEM. Transfected cells were then cultured at 37 °C for three days, washed three times with DMEM without FBS, and incubated with DMEM containing 5 μg/mL of trypsin (Sigma-Aldrich, St. Louis, MO, USA). Viruses were harvested 6 h thereafter by freeze-thawing transfected cells and culture supernatant three times, and stored at −80 °C as P0 virus. To propagate the virus, Vero cells were seeded onto a 35 mm dish (VIOLAMO, Osaka, Japan), cultured overnight, washed three times with DMEM without FBS, and inoculated with 2 mL of P0 virus. After 2–3 h, wells were washed once with DMEM without FBS, and filled with 2 mL of DMEM containing 10 μg/mL of trypsin and 0.3% tryptose phosphate broth (Sigma-Aldrich, St. Louis, MO, USA). Cells were then incubated at 37 °C for three or four days until cytopathic effects (CPEs) were observed. Harvested P1 viruses were passaged one more time on Vero cells to generate the P2 viruses, which were stored at –80 °C until use.

### 2.4. Animal Studies

Gnotobiotic newborn piglets, which are highly susceptible to infectious agents, were obtained from four specific pathogen-free sows, following guidelines for the proper conduct of animal experiment at National Institute of Animal Health (17-061, 5 December, 2017, NIAH Animal experiment committee).

#### 2.4.1. Experimental Infection in 7-Day-Old Gnotobiotic Piglets

Twelve seven-day-old piglets were randomly assigned to orally receive CV777-niah (10^6.3^ TCID_50_/head, *n* = 3), its recombinant form, rPEDVGFP-CV777 (10^5.6^ TCID_50_/head, *n* = 5), and the S-chimeric strain, rPEDVGFP-S-OKN1 (10^4.7^ TCID_50_/head, *n* = 4). Fecal samples were collected from each piglet every day from 0–5 days post-inoculation (DPI) and every 2–4 days thereafter. Sera were collected from each piglet every 2–3 days from 0 to 7 DPI and every 3–4 days thereafter. All animals were monitored for clinical signs and virus shedding until 28 DPI. Viral RNA was extracted from 10% fecal suspensions and sera using QIAmp Viral RNA Mini Kit (Qiagen, Hilden, Germany), and titers were quantified by real-time reverse-transcription quantitative PCR using Takara One Step PrimeScript RT-PCR Kit (Takara Bio, Shiga, Japan) as described previously [26].

#### 2.4.2. Experimental Infection in Five-Day-Old Gnotobiotic Piglets

Eighteen five-days-old piglets were randomly assigned to orally receive the S-chimeric strain, rPEDVGFP-S-OKN1 (10^5.8^ TCID_50_/head, *n* = 6), the S1-chimeric strain, rPEDVGFP-S1-OKN1 (10^5.8^ TCID_50_/head, *n* = 6), or DMEM with no anything as mock control (*n* = 6). Feces and sera were collected as described above. Three piglets from each group were euthanized at 2 DPI, and remaining piglets were monitored for clinical signs and virus shedding until 28 DPI. On necropsy, small and large intestines were collected, along with heart, lung, kidney, liver, spleen, tonsil, trachea, muscle, stomach, and mesenteric lymph node. Viral RNA was extracted from 10% fecal suspensions, sera, and 10% tissue homogenates as described above. Virus shedding in feces and sera, and virus distributions in tissues at 2 DPI were quantified by real-time reverse-transcription quantitative PCR of the N gene as described above.

### 2.5. Virus Isolation and Sequencing

To isolate viruses from PEDV-inoculated piglets, feces collected at peaks virus shedding were suspended at 10%, and inoculated into Vero cells. The inoculum was removed after 1 h at 37 °C in a humidified atmosphere with 5% CO_2_. Cells were then washed three times with DMEM without FBS, and filled with DMEM containing 10 μg/mL trypsin and 0.3% tryptose phosphate broth. After two or three days at 37 °C, CPEs such as cell fusion and syncytium formation were assessed by microscopy, along with GFP expressions.

The genomes of viruses isolated from PEDV-inoculated piglets were sequenced on an Ion Torrent PGM (Thermo Fisher Scientific, Carlsbad, CA, USA) as described previously [24].

### 2.6. Antibody Detection by Immunofluorescence Assay

Confluent monolayers of Vero cells grown in 96-well plates (Sumitomo Bakelite, Tokyo, Japan) were inoculated with 10^2.5^ TCID_50_ OKN-1/JPN/2013 in 100 μL, and incubated at 37 °C in a humidified atmosphere with 5% CO_2_. Inocula were removed after 18 to 24 h. Subsequently, cells were washed with PBS once, and fixed with cold 70% acetone (FUJIFILM Wako Pure Chemical, Osaka, Japan) for 10 min. Plates were then air dried, sealed and stored at –20 °C until probed with 50 μL sera serially diluted two-fold from 1:20 to 1:1280 in PBS. Sera were removed after 1 h at 37 °C, and plates were washed 5 times with PBS. Wells were then reacted for 1h at 37 °C with 50 μL/well of 1:50 goat anti-swine IgG (H + L) conjugated to fluorescein isothiocyanate (Jackson ImmunoResearch, West Grove, PA, USA), washed five times with PBS, an examined for PEDV-specific cytoplasmic staining under a fluorescent microscope. Antibody titers are reported as the reciprocal of the highest dilution that produced clear, specific cytoplasmic staining.

## 3. Results

### 3.1. Recovery of Recombinant PEDVs Derived from CV777

**Figure 1:**
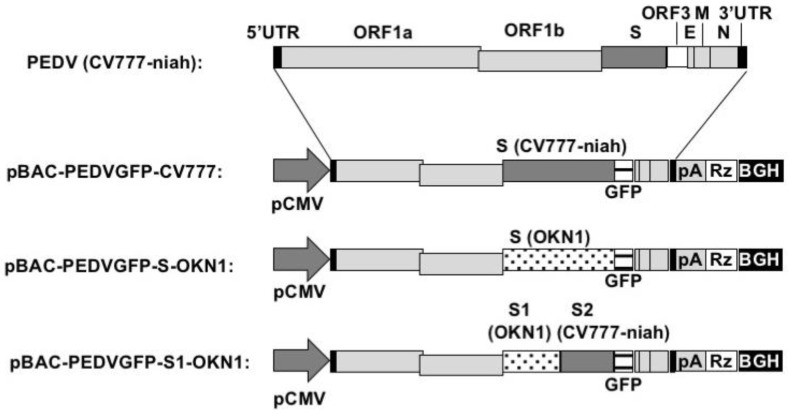
Structure of the PEDV genome from CV777 maintained in National Institute of Animal Health (top) and bacterial artificial chromosome cDNA (2nd, 3rd, and bottom). Black boxes represent 5’ and 3’ untranslated regions. Light gray boxes represent ORF 1a, ORF1b, E, M, and N, while dark gray and white boxes represent S and ORF3, respectively. pCMV, represents cytomegalovirus promoter; pA, polyA tail; Rz, HDV ribozyme; BGH, BGH termination and polyadenylation sequence.

To evaluate virulence due to the *S* gene, we first attempted to establish an infectious clone (pBAC-PEDV-CV777-FL) carrying a whole genome of the CV777-niah strain except for 3’ end of the S gene by using a BAC system. The CV777 cDNA was assembled in the BAC under the control of the cytomegalovirus promoter and flanked at the 3’ by 25 bp of poly(A), a hepatitis D virus (HDV) ribozyme, and a bovine growth hormone (BGH) sequence (Figure 1). The *ORF3* gene was then replaced with *GFP* gene driven by the ORF3 transcription regulatory sequence to generate pBAC-PEDVGFP-CV777. Two chimeric clones, pBAC-PEDVGFP-S-OKN1 and pBAC-PEDVGFP-S1-OKN1, were subsequently generated by replacing the *S* and *S1* genes in pBAC-PEDVGFP-CV777, respectively, with those of the OKN-1/JPN/2013 strain. The P2 viruses isolated in Vero cells were confirmed the CPEs, the same as GFP expressions by microscopy. Moreover, genomic sequences of the P2 viruses were confirmed to be identical to those of the sources.

### 3.2. Experimental Infection in Seven-Day-Old Gnotobiotic Piglets

Newborn piglets inoculated with the CV777-niah and its recombinant form, rPEDVGFP-CV777 developed subclinical to mild diarrhea and anorexia 3–7 DPI (Table 2). In contrast, the piglets inoculated with the S-chimeric strain, rPEDVGFP-S-OKN1 developed severe watery diarrhea and eventually anorexia, lethargy, severe dehydration, weight loss, and astasia 2–7 DPI. However, these animals recovered beginning at 14 DPI and ultimately survived to the end of the experiment. Immunofluorescent assay for antibodies confirmed that all PEDV-inoculated piglets were infected (Table 2).

**Figure 2:**
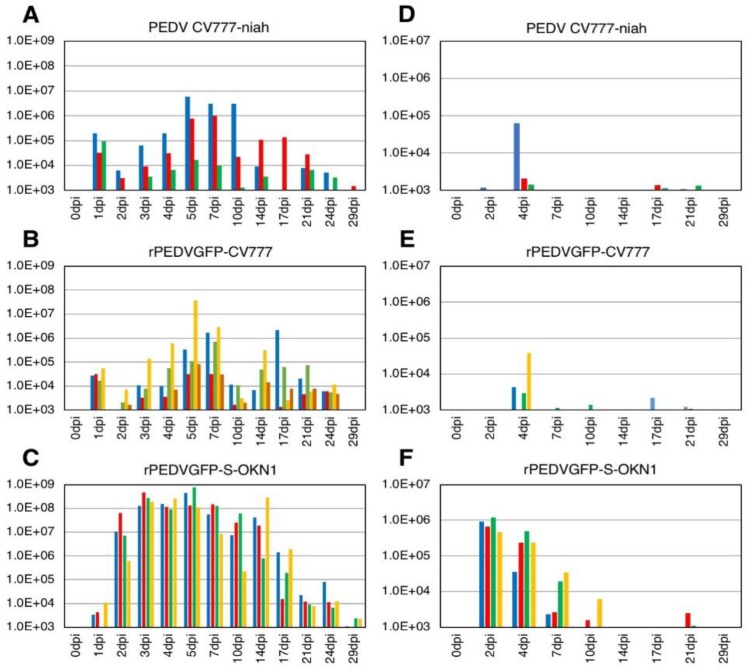
Virus shedding in feces (**A**–**C**) and sera (**D**–**F**) in piglets inoculated with the parental strain CV777-niah (**A**,**D**), its recombinant form, rPEDVGFP-CV777 (**B**,**E**), and the S-chimeric strain, rPEDVGFP-S-OKN1 (**C**,**F**). Data are genome equivalents/mL in individual animals indicated in different colors, as quantified at each time point by a real-time reverse-transcription quantitative PCR for the *N* gene.

**Figure 3:**
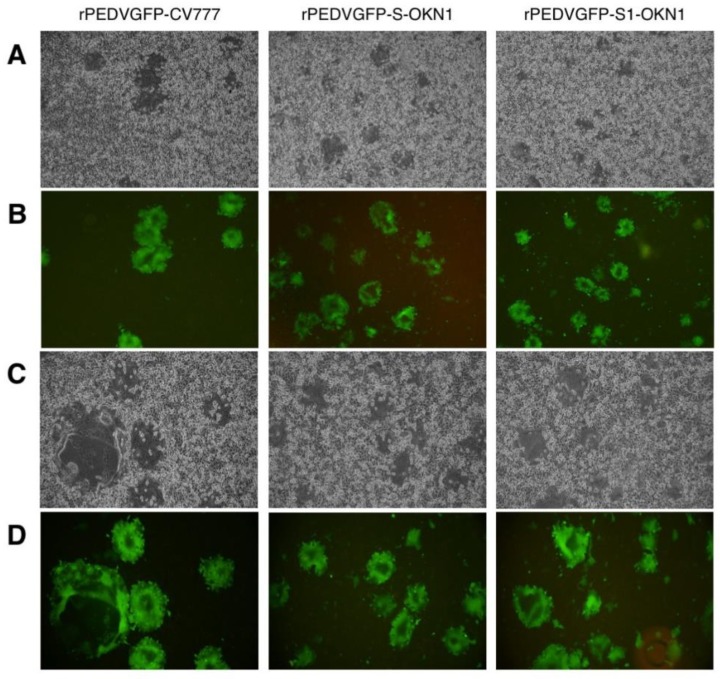
Cytopathic effects (**A**,**C**) and GFP expressions (**B**,**D**) by a microscopic observation of recombinant PEDVs, rPEDVGFP-CV777, rPEDVGFP-S-OKN1, and rPEDVGFP-S1-OKN1 isolated from feces collected at peak virus shedding. (**A**,**B**): Magnification, 40×, (**C**,**D**): magnification, 100×.

Piglets inoculated with the S-chimeric strain exhibited peak viral shedding of 10^8^–10^9^ genome equivalents (GE)/mL in feces at 4–7 DPI. Shedding was sustained at high levels for approximately two weeks (Figure 2). Piglets inoculated with the CV777-niah and the recombinant strain reached transient peak shedding of 10^4^–10^6^ and 10^4^–10^7^ GE/mL in feces at 5–7 DPI. Viral RNAs was detected at 10^5^–10^6^ GE/mL in the sera 2–4 days after inoculation with the S-chimeric strain (Figure 2). Viral RNA also peaked at 10^3^–10^5^ GE/mL four days after infection with the CV777-niah and the recombinant strain. The recombinant and chimeric viruses collected from feces of infected piglets at the peak virus shedding elicited GFP expressions and CPEs in Vero cells, as assessed by microscopy, confirmed that the viruses are maintained and propagated in piglets, as suggested by sequencing (Figure 3).

### 3.3. Experimental Infection in Five-Day-Old Gnotobiotic Piglets

Piglets inoculated with the rPEDVGFP-S-OKN1 and rPEDVGFP-S1-OKN1 strains exhibited acute severe watery diarrhea from 1 DPI. At 2–4 DPI, these animals additionally developed anorexia, lethargy, severe dehydration, weight loss, and astasia (Table 3). Finally, all animals died by 7 DPI. On the other hand, piglets infected with culture medium did not develop clinical signs, and survived to the end of the experiment.

**Figure 4:**
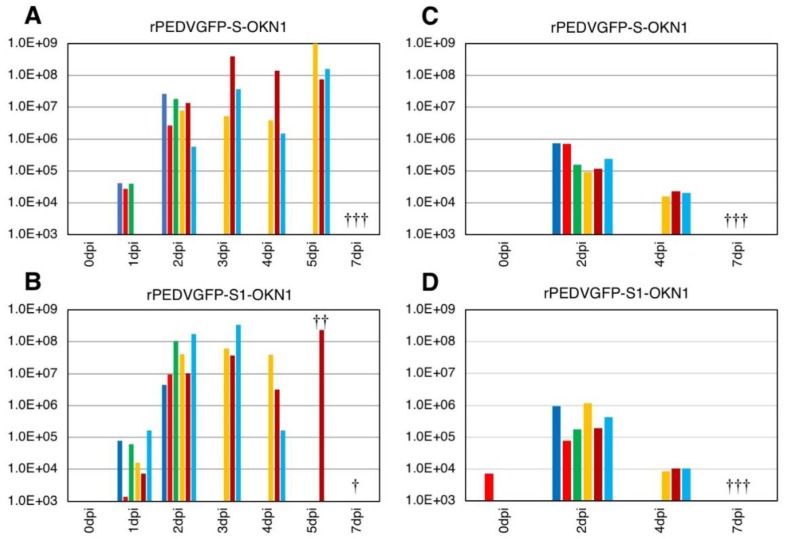
Virus shedding in feces (**A**,**B**) and sera (**C**,**D**) from piglets inoculated with the S-chimeric strain, rPEDVGFP-S-OKN1 (**A**,**C**), the S1-chimeric strain, rPEDVGFP-S1-OKN1 (**B**,**D**). Data are genome equivalents/mL in individual animals indicated in different colors as quantified at each time point by a real-time reverse-transcription quantitative PCR for the *N* gene. Three piglets from each group were euthanized at two days post-infection. The symbols (†) in graph mean death of individual piglets during observation period.

**Figure 5:**
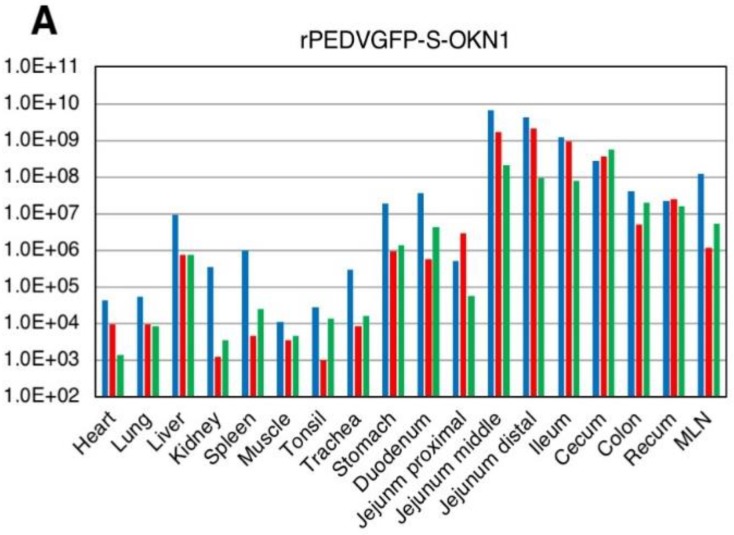

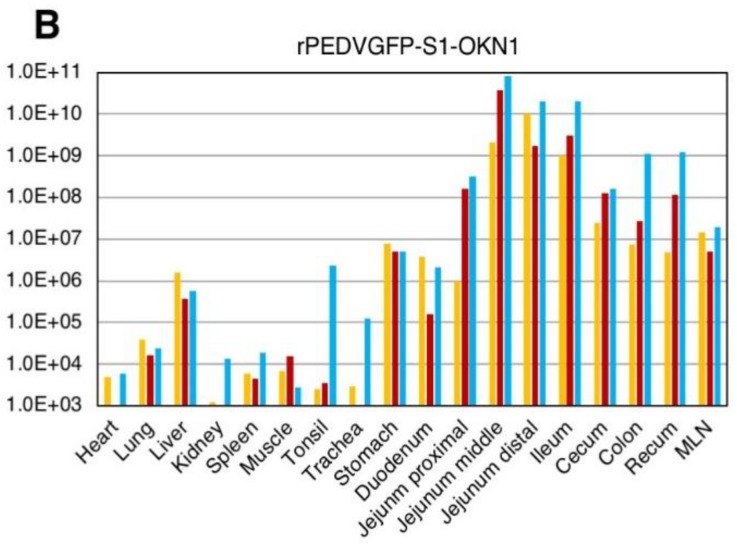
Virus distributions in various tissues collected from piglets inoculated with the-S chimeric strain, rPEDVGFP-S-OKN1 (**A**) and the S1-chimeric strain, rPEDVGFP-S1-OKN1 (**B**). Data are genome equivalents/mL in individual animals indicated in different colors as quantified at each time point by a real-time reverse-transcription quantitative PCR for the *N* gene.

Viral RNA titers in feces and sera are plotted in Figure 4. Fecal samples collected 1–2 DPI with the S- and S1-chimeric strains tested positive for PEDV by PCR, with peak viral shedding at 10^7^–10^9^ GE/mL within 3–5 DPI. Those titers were sustained at high levels until death. Viruses collected from feces at the peak shedding of piglets infected with the S- and S1-chimeric strains were confirmed by virus isolation and sequencing to be derived from inocula (Figure 3). Viral RNAs were also detected at 10^4^–10^6^ and 10^5^–10^7^ GE/mL in sera 2 DPI with the S- and S1-chimeric strains, respectively. In piglets infected with the S-chimera viruses were mainly detected at 10^7^–10^10^ GE/mL in intestinal tissues from the stomach to the rectum, as well as in mesenteric lymph node (Figure 5). In animals infected with the S1-chimera, viruses at 10^7^–10^12^ GE/mL were widely distributed in the small and large intestines and in mesenteric lymph node. In contrast, fecal, sera, and tissue samples from mock-infected piglets tested negative on PEDV-specific PCR.

## 4. Discussion

In comparison of whole genomes between the CV777-niah and the reference CV777 (GenBank accession no. AF353511), we found 11 nucleotide difference in 5’ UTR, ORF1a/1b, spike, and nucleocapsid, two deletions (1 or 4 nucleotides) in 5’ UTR and 52 nucleotides deletion in Spike/ORF3 junction (Table 1). Especially, 52 nucleotides deletion in Spike/ORF3 junction in the CV777-niah can be predicted to result in the removal of seven amino acids from C-terminus of spike protein and loss of ORF3 protein expression as reported in a previous study [37]. In addition, we generated the recombinant and chimeric PEDV strains which replaced their *ORF3* genes with *GFP* genes and, hence, these strains can also be expected to be complete loss of their ORF3 functions. Piglets inoculated with the CV777-niah and its recombinant form showed similar symptoms and patterns of fecal virus shedding, and survived throughout the experiment. In contrast, Pensaert et al. reported that pathogenicity of piglets inoculated with the prototype stain CV777 is identical to those observed by the North American PEDV stains [38]. The difference of pathogenicity between the CV777-niah and the prototype CV777 might be involved in nucleotide change by cell adaptation, especially loss of ORF3 function, because there are previous reports that ORF3 differentiation through cell adaptation is associated with reduced virulence [39,40,41].

In the first experiment, all seven-day-old gnotobiotic piglets inoculated with the S-chimeric PEDV strain, rPEDVGFP-S-OKN1 (10^4.7^ TCID_50_/head), exhibited extreme debilitating at 2–7 DPI, including severe watery diarrhea, anorexia, lethargy, severe dehydration, weight loss, and astasia, but ultimately survived until 28 DPI (Table 2). In contrast, in the second experiment, three five-day-old gnotobiotic piglets inoculated with the same S-chimeric strain (10^5.8^ TCID_50_/head) died at 7 DPI after exhibiting anorexia, lethargy, severe dehydration, weight loss, and astasia in addition to severe watery diarrhea (Table 3). Moreover, two of three five-day-old gnotobiotic piglets inoculated with the S1-chimeric strain, rPEDVGFP-S1-OKN1 (10^5.8^ TCID_50_/head) died at 5 DPI, and the other died at 7DPI with similar clinical signs as piglets inoculated with the S-chimeric strain. The discrepancy in mortality of piglets with the S-chimeric strain suggests to be associated with the difference of viral titers inoculated with piglets or the difference of age in onset of two experiment.

Piglets inoculated with the S- and S1-chimeric PEDVs showed similar patterns of acute virus shedding in feces and sera from 1–2 DPI (Figure 2 and Figure 4). Moreover, necropsy at 2 DPI indicated that viruses were predominantly restricted to the intestinal tract from jejunum to rectum, and to mesenteric lymph node (Figure 5). Previously, we reported that six-day-old colostrum-deprived piglets inoculated with OKN-1/JPN/2013, which is the source of S and S1 fragments inserted into chimeric PEDVs, showed severe clinical signs such as dehydration, weight loss, acute viral shedding in feces and sera from 1–2 DPI, and high virus titers in small and large intestines and mesenteric lymph node, and ultimately died by four days post-infection [26]. Thus, the chimeric strains with *S* and *S1* genes from OKN-1/JPN/2013 appear to reproduce its virulence. Consequently, our data strongly suggests that the *S* gene, especially the S1 subunit, is an important factor of severe symptoms and mortality in piglets. The difference of pathogenicity in piglets using these recombinant viruses might be caused by the function of spike protein in virus entry or process of budding. In future, we would perform in vitro assay using these viruses to elucidate the mechanism.

Additionally, using reverse genetics, Hou et al. found that deletion of 197 amino acids from the N-terminal domain of the S1 subunit attenuated the pathogenicity of a highly virulent PEDV [32]. This results strongly supports the hypothesis that the S1 subunit is an important determinant of virulence, especially since we now demonstrate the opposite effect, in which virulence is conferred via S1 from a highly virulent strain upon an avirulent strain CV777-niah. In contrast, Wang et al. reported that the *S* gene is necessary but not sufficient for the virulence, based on experimental infections with various chimeric PEDVs [33]. However, these experiments were based on conventional piglets, while we and Hou et al. used gnotobiotic piglets, so results may not be directly comparable, especially since there were clear differences in pathogen susceptibility between conventional and gnotobiotic piglets. In any case, further studies based on reverse genetics are needed to clarify the function and role of other PEDV genes.

In summary, we generated recombinant PEDVs derived from the CV777-niah maintained in cell culture, as well chimeric PEDVs in which the original *S* gene is replaced with a virulent form by reverse genetics. The resulting S- and S1-chimeric strains caused severe clinical signs or high mortality in highly susceptible gnotobiotic piglets of different ages, reproducing the pathogenicity of OKN-1/JPN2013 as we described previously. Collectively, the data indicate that the S1 subunit is an important determinant of PEDV virulence. We will, thus, attempt to further map the sequences in the S1 subunit that are critical to virulence.

## Figures and Tables

**Table 1:** Nucleotide and amino acid differences by comparison of the complete genomes between CV777 maintained in National Institute of Animal Health and reference CV777 (GenBank accession number AF353511).

Region	Position of Nucleotides in Reference CV777	Nucleotide in Reference CV777	Amino Acid in Reference CV777	Nucleotide in CV777-Niah	Amino Acid in CV777-Niah
5’-UTR	72	a		Deletion	
5’-UTR	82–85	tcct		Deletion	
ORF1a/1b	1667	t	A	c	A
ORF1a/1b	6052	t	V	c	A
ORF1a/1b	6593	c	H	t	H
ORF1a/1b	6630	t	F	c	L
ORF1a/1b	10,542	g	V	a	I
ORF1a/1b	11,887	g	G	a	D
ORF1a/1b	12,257	t	D	c	D
Spike	22,145	c	S	t	L
Spike	23,323	g	D	a	N
Spike	24,461	a	N	c	T
Spike/ORF3	24,765–24,816	*		Deletion	
Nucleocapsid	26,940	c	N	t	N

Gray shadows represent non-synonymous substitutions by comparison of amino acids between CV777-niah and reference CV777. * 52 nucleotide deletion at Spike/ORF3 junction: TTTTGAAAAGGTCCACGTGCAGTG**A****TG**TTTCTTGGACTTTTTCAATACACGA. Stop codon of spike and start codon of ORF3 indicate with underline and bold, respectively.

**Table 2:** Clinical signs and geometric mean titers of antibodies at 28 days post-infection in piglets inoculated with CV777 maintained in National Institute of Animal Health, CV777 recombinant, and S-chimeric PEDV strains.

Inoculum	n	% Mortality	Clinical Signs						Antibodies (GMT)
			Diarrhea	Anorexia	Lethargy	Dehydration	Weight Loss	Astasia	
CV777-niah	3	0 (0/3)	Mild	+	-	-	-	-	23.8
rPEDVGFP-CV777	5	0 (0/5)	Mild	+	-	-	-	-	44.9
rPEDVGFP-S-OKN1	4	0 (0/4)	Severe	+	+	+	+	+	160

**Table 3:** Clinical signs in piglets inoculated with S-chimeric and S1-chimeric PEDV strains, and culture medium (Mock).

Inoculum	n ^a^	% Mortality	Death at Days Post-Inoculation	Clinical Signs					
				Diarrhea	Anorexia	Lethargy	Dehydration	Weight Loss	Astasia
rPEDVGFP-S-OKN1	6	100 (3/3)	3/3 (7 DPI)	Severe	+	+	+	+	+
rPEDVGFP-S1-OKN1	6	100 (3/3)	2/3 (5DPI) 1/3 (7DPI)	Severe	+	+	+	+	+
DMEM (Mock)	6	0 (0/3)	0/3 (28 DPI)	-	-	-	-	-	-

^a^ Three piglets from each group were euthanized at two days post-infection, and the remaining piglets were monitored for clinical signs until 28 days post-inoculation.
